# Evaluating the capacity of paired comparison methods to aggregate rankings of separate groups

**DOI:** 10.1007/s10100-023-00839-3

**Published:** 2023-02-12

**Authors:** Éva Orbán-Mihálykó, Csaba Mihálykó, László Gyarmati

**Affiliations:** Faculty of Information Technology, University of Pannonia, Egyetem u. 10, Veszprém, H8200 Hungary

**Keywords:** Aggregation, AHP, Bradley–Terry model, Evaluation of sports tournaments, Maximum likelihood estimation, Paired comparison, Thurstone method, Weighted LLSM

## Abstract

Ranking and rating methods have outstanding significance in sports, mainly due to their capacity to predict results. In this paper we turn to their capacity to aggregate separate groups’ rankings based on a small piece of information. We investigate under which conditions two or more separate groups can be trustworthily interwoven applying Thurstone motivated methods and an AHP based method. A theorem is proved which guarantees adequate unified ranking based on some links between the groups. We also analyse the robustness of the results.

## Introduction

Evaluating objects on the basis of paired comparisons is a frequently used technique in several fields of life. The fields of applications include psychology (Sung and Wu 2018), marketing (Leung and Mo 2019), education (Wyatt-Smith et al. 2020), products’ evaluation (Čubrić et al. 2019), or management (Esangbedo et al. 2021). Moreover, the method can be applied in sports: the results of matches can be considered as the results of paired comparisons. Recent publications have explored the application of the method to sports, see, for example (Bozóki et al. 2016; Baker and McHale 2017) to tennis, (Arntzen and Hvattum 2021) to football, (Anderson 2014) to racing sports, Araki et al. (2019) to sumo, (Csató 2021b; Orbán-Mihálykó et al. 2022) to handball, (Csató 2017; Hankin 2020) to chess e.t.c. The main questions are ranking and rating, moreover the predicting capabilities are in the centre of the research. Comparisons of some methods based on their capacity to predict the results are contained in Lasek and Gagolewski (2021), Arntzen and Hvattum (2021) with detailed literature.

Aggregation of individual preferences is in the focus of the research, see for example (Duleba and Szádoczki 2022) and the detailed references in. Applying stochastic methods, our paper considers the set of match results as a data set, and aggregation concern the different subgroups of teams. A recent line of research on paired comparisons-based ranking aims to determine the optimal set of comparisons (Gyarmati et al. 2022; Szádoczki et al. 2022a, b). From a sporting perspective, the problem is to find the set of matches to be played if the number of matches is fixed (Sziklai et al. 2022). These results can be important when the organizers fix the rules of tournaments.

In this paper, we scrutinize the methods from another point of view: we assess them based on their quality when we use them to aggregate separate groups. It is also an important property, because sports tournaments frequently have two stages: in the first stage the players (teams) compete in separate groups, then a knockout stage follows: the loser of the match is eliminated from the tournament. Lots of information is available from the matches in the groups, but how can we set up a unified ranking containing all players in the groups? How can we capitalize on the available information? What is the piece of information we need, and how much is enough to set up a reliable unified ranking in the case of different paired/pairwise comparison methods? These are the main questions of the present paper. Although the example investigated is from sports, the same questions are relevant in other areas, too.

In Orbán-Mihálykó et al. (2019a), the authors investigated Thurstone method and proved a sufficient condition under which the results can be uniquely evaluated. This statement was extended for a large set of distributions, as Orbán-Mihálykó et al. (2019b) presented. Nevertheless, this sufficient condition proved to be too strict during the aggregation. In this paper its generalization is presented and applied aggregating separate groups in the case of both Thurstone and Bradley–Terry models.

The paper is organized as follows: after a short review of the literature (Sect. 2), in Sect. 3, we present a short summary of the applied methods. In Sect. 4 we provide a new statement which proves to be useful in interweaving separate groups. In Sect. 5, the results of the unified ranking of the groups of EHF Women’ Champions League are presented. In Sect. 6, the robustness of the aggregation is analyzed. Finally, a short conclusion closes the paper.

## A short review of the literature

The method most frequently used to comparisons in pairs is AHP (Analytic Hierarchy Process), which was elaborated by Saaty (1977, 1980). His works have thousands of citations. Although the method was elaborated to evaluate opinions, its potential for evaluating sports results has been already presented (Csató 2013; Bozóki et al. 2016). The starting point is a pairwise comparison matrix. Its elements express the ratio of the strengths of the objects to evaluate. These elements are usually 1, 3, 5, 7, 9 and their reciprocals. In the case of sports, sometimes another rate, the ratio of wins and lost matches is used. However, based on a low number of matches played against each other it is difficult to find a trustworthy quantity for the mentioned ratio. Moreover, for a pairwise comparison matrix, the most frequently used evaluation method is the principal eigenvector method. It is easy to perform, but it requires all the elements in the matrix. This condition does not hold if we concatenate separate groups.

In case of incomplete comparisons (some teams do not play with some others), in Bozóki et al. (2010) the authors present two alternative evaluation methods, called logarithmic least squares method (LLSM) and incomplete eigenvector method (IEM), but the construction of the comparison matrix still cannot be avoided.

The second group of paired comparison methods, probabilistic methods work on different principles. They operate with latent random variables. The actual values of the difference of these random variables determine the result of the comparison. The expectations of the random variables determine the ranking and ratings. The method was elaborated by Thurstone (1927), who applied it to evaluate subjective opinions in psychology. The concept of stochasticity is not far from reality in sports. The distribution of the differences of the latent random variables might be chosen from a wide set of functions, the axiomatic properties of the methods remain the same (see Orbán-Mihálykó 2019b).

Thurstone applied two options (better and worse) and assumed Gauss distributed latent random variables.

In Bradley and Terry (1952), the authors supposed logistic distribution for the differences allowing two options. Their model was generalized for ties in Rao and Kupper (1967). In Stern (1992), it has been shown for two options that the Bradley–Terry model and the Thurstone model are the limits of a certain model. The number of options can be increased. In Agresti (1992) the least squares methods, in Orbán-Mihálykó et al. (2019a) the maximum likelihood estimation (ML) is applied for parameter estimations. The hinge of the maximum likelihood estimation is the existence of the maximal value and the uniqueness of its argument. For this, allowing two options, Ford (1957) contains necessary and sufficient condition in the case of logistic distribution. However, this condition is generalized for three options in Davidson (1970), but this condition is only a sufficient condition, but not necessary. A different condition is given in Orbán-Mihálykó et al. (2019b) for arbitrary number of options and general distributions, which is again a sufficient but not necessary condition. However, these conditions are too strict in the case of aggregation, they are not always satisfied when concatenating different groups.

The third group of evaluation methods is the set of Elo motivated evaluations (Berg 2020). The Elo-method is the generally accepted method in chess, it is used for the official rating of chess players (Elo 1978). While the previous methods handle the set of objects to be evaluated as a complex system, in the Elo-motivated methods the strengths of objects change in pairs. If two objects are compared (two players play a match), then their Elo-points will change in the function of the result of the match played and the differences in their strengths, but the others’ remain the same. The method is local method in the following sense: strengths are changing step by step and during one step only those teams’ strengths change which play the match, all the others’ Elo-points are left untouched. An excellent survey on Elo-based methods is in Aldous (2017). These methods are used in neural networks, too. The comparisons of several Elo-methods concerning their predicting properties are contained in Lasek and Gagolewski (2021).

It is easy to see that even though these methods can be advantageous for predictions, their interweaving properties might be unfavourable due to the "local" feature. We mention that an Elo-based approach is used for the FIFA Ranking of women’s national teams (Van Eetvelde and Ley 2019) and for the FIFA Ranking of men’s national teams since 2018 (FIFA 2018). From axiomatic point of view the paper (González-Díaz et al. 2014) contains a detailed comparison of the frequently used methods for two options. It concludes that the Bradley–Terry model with ML estimation and the generalized row sum method elaborated by Chebotarev (Chebotarev 1994) are the most favourable from axiomatic point of view. The paper (Orbán-Mihálykó et al. 2019b) has proved that the Thurstone motivated methods behave similarly to each other. We deal with them in this paper investigating their interweaving properties.

## A short summary of the applied methods

### AHP with weighted LLSM (AL)

Since in the group stages of the tournaments the teams usually play one or two matches with all the others, we have decided to construct the AHP matrix as follows.

Let *n* be the number of the players to evaluate and let them be denoted by $1,2,\ldots ,n$. Let the AHP matrix be $B=(b_{ij})$ $i=1,\ldots ,n$; $j=1,\ldots ,n$. During the $m^{th}$ match between *i* and *j* $b_{ij}^{(m)}=3,$ if *i* is better than *j* (*i* beats *j*), $b_{ij}^{(m)}=1,$ if the result is draw and $b_{ij}^{(m)}=1/3,$ if *i* is worse than *j* (*j* beats *i*). This is a very special case of AHP technique, the scale belonging to a single match is reduced for 3 possible values. As in this paper the stochastic models apply 3 options, we think, that this might be the appropriate model to compare. In case of *k* matches between the teams *i* and *j*, take the geometric mean: $b_{ij}=\root k \of {\prod \limits _{m=1}^{k}b_{i,j}^{(m)}},$ and $b_{i,j}^{(m)}$ is constructed on the basis of the $m^{th}$ match. If the player *i* does not play any match with the player *j*, then the element $b_{ij}$ is not defined, its place remains empty in the matrix. If there is at least one match between the teams *i* and *j*, it can be easily seen that $b_{ij}=\frac{1}{b_{ji}}.$ The LLSM method evaluates the above PC matrix by logarithmic least squares method as follows (Bozóki et al. 2010): minimize the function

1 $$\begin{aligned} H({\underline{w}})=\sum _{I}\left( \log (b_{i,j})-(\log (w_{i})-\log (w_{j}\right) ))^{2} \end{aligned}$$

under the conditions 0$<w_{i},i=1,2,\ldots ,n,\sum _{i=1}^{n}w_{i}=1.$ The set *I* contains all the pairs for which there is at least one comparison. In Bozóki et al. (2010) the authors prove that necessary and sufficient condition of the existence and uniqueness of the optimization problem is the connectedness of the graph of comparisons, as well as in the case of IEM. The graph of comparisons $G_{c}$ is defined as follows: the vertices are the players and there is an edge between two vertices if there is a match between the players.

This method does not contain the number of matches between the teams. In our examples, these numbers can be 1 or 2, depending on the number of cancelled matches caused by pandemic. To keep this information, we introduce weights in the objective function, similarly to in Csató and Tóth (2020), Petróczy (2021). Consequently, (1) is replaced by

2 $$\begin{aligned} WH({\underline{w}})=\sum _{I}N_{i,j}\left( \log ( b_{i,j})-(\log (w_{i})-\log (w_{j}\right) ))^{2} \end{aligned}$$

where $N_{i,j}$ is the number of matches between players *i* and *j*. The objective function (2) is maximized under the conditions 0$<w_{i},i=1,2,\ldots ,n,\sum _{i=1}^{n}w_{i}=1.$ One can check that it can be uniquely maximized if and only if the objective function (1) can.

### Thurstone motivated methods (TMM)

Let us consider the performances of the teams as random variables denoted by $\xi _{i}$, $i=1,2,3,\ldots ,n$. We allow three options as a result of a match: win, tie and defeat. Now $\xi _{i}-\xi _{j}=m_{i}-m_{j}+\eta _{i,j},$ where $\eta _{i,j}$ are supposed to be independent, identically distributed random variables with the cumulative distribution function *F*. *F* is a general three times differentiable c.d.f. with $0<F(x)<1,$ and it has a symmetrical, strictly log-concave probability density function. We will use the notation ${\mathbb {F}}$ for this subset of c.d.f.’s. If *F* is the standard normal (Gauss) c.d.f., then the model is the generalization of the Thurstone model (TH). If *F* is the logistic c.d.f., then Bradley–Terry model with tie is used (BT).

The probabilities of the results of team *i* and *j* can be expressed as follows:

3 $$\begin{aligned}{} & {} p_{i,j,1}=P(\text {team }i\text { is defeated by team }j)=F(-d-(m_{i} -m_{j})) \end{aligned}$$

4 $$\begin{aligned}{} & {} p_{i,j,2}=P(\text {the result is draw})=F(d-(m_{i}-m_{j}))-F(-d-(m_{i}-m_{j})) \qquad \end{aligned}$$

5 $$\begin{aligned}{} & {} p_{i,j,3}=P(\text {team }i\text { wins over team }j)=1-F(d-(m_{i}-m_{j})). \end{aligned}$$

Here the parameter $0<d$ assigns the boundary of the tie.

Let *A* be a three dimensional data matrix with sizes *n*x*n*x3 and with elements $A_{i,j,k}$ $i=1,2,\ldots ,n,$ $j=1,2,\ldots ,n,$ $k=1,2,3.$ $A_{i,j,k}$ is the number of matches when team *i* has result *k* against team $j,i<j$; $k=1$ stands for defeats, $k=2$ for draws, and $k=3$ for wins. If $j\le i,$ then let $A_{i,j,k}$ =0, $k=1,2,3$. The probability of the results given by data matrix *A* in the function ${\underline{m}}=(m_{1},m_{2},\ldots ,m_{n})$ and 0 < *d*,  supposing the independence of the sample elements, is

6 $$\begin{aligned} L(A|{\underline{m}},d)=\prod \limits _{k=1}^{3}\prod \limits _{i=1}^{n-1} \prod \limits _{j=i+1}^{n}p_{i,j,k}^{A_{i,j,k}}. \end{aligned}$$

*L* is called likelihood function. The maximum likelihood estimation of parameters ${\underline{m}}$ and 0 < *d*, denoted by $\widehat{{\underline{m}}}$ and ${\hat{d}}$, is the $n+1$ dimensional argument, where the function *L* reaches its maximal value (Eliason 1993).

The crucial element of the estimation process is to ensure the existence and uniqueness of the maximizer. If the maximum of the likelihood function does not exist or the argument is not unique, the method does not work. If the maximum was not reached, the method can not provide evaluation. If the maximum was not unique, the evaluation is not definite. Instead of (6), often its logarithm (log-likelihood) is maximized. As (6) is greater than 0, the logarithm can be taken, and the multiplications become sums. Due to the strictly monotone increasing property of the logarithm function, (6) has unique maximizer if and only if its logarithm has.

The problem of the existence and uniqueness is not usually observed in the case of TMM. If it is, the justification is often inaccurate. The paper (Arntzen and Hvattum 2021) makes the following remark on page 457: “As the likelihood function is convex, Newton’s method can be applied to maximize the likelihood once the gradient and Hessian of the function has been derived”. However, the convex property is connected to minimum (see $f(x)=x^{2}$) and the ML estimation seeks a maximum, therefore, the property convex must be a typographical error. The Newton method can in fact be applied to find the maximum numerically in case of concave functions, if the maximum exists. But if we have a concave function (see, for example $f(x)=\textrm{log}(x)$), it is monotone increasing and does not have a maximal value on $(0,\infty )$. If we investigate a concave function on a finite and closed interval, the maximal value exists, but the uniqueness of the maximizer is guaranteed only in case of strictly concave functions (Eliason 1993).

The boundedness of the parameters and the strictly concave properties are far from being obvious in the case of the logarithm of the likelihood functions. They hold only in special cases concerning coefficients $d_{ij}$ in Arntzen and Hvattum (2021). As a simple example, take $d_{i,j}=0,$ for *all* pairs (*i*, *j*). In this (mathematical) case the likelihood function is concave, the maximum exists but its argument is not unique. Furthermore, an example, when the maximum does not exist: take $d_{1,1}=1$ and $d_{i,j}=0$ in all other cases. More sophisticated examples can also be constructed (see Orbán-Mihálykó 2019a), but the above-mentioned examples may be sufficient to convince the reader about the problems. And the problems are even more conspicuous if we want to link separate subgroups.

Allowing two options, win and loss, first (Ford 1957) formulated a condition for the existence and uniqueness of the maximizer of the likelihood function in the case of the Bradley–Terry model, which was generalized by Davidson allowing ties. The statement motivated by Davidson (1970) can be proved for general $F\in {\mathbb {F}}$ in the following form:

#### Theorem 1

Let us suppose that there is at least one tie, i.e. there exists a pair (*i*, *j*) for which

7 $$\begin{aligned} 0<A_{i,j,2}. \end{aligned}$$

Suppose that every nonempty partition of the objects 1,2,...,n, *S* and its complement ${\overline{S}}$, there is at least one object $i_{1}\in$ *S* and $j_{1}\in {\overline{S}}$, $i_{2}\in S,$ $j_{2}$ $\in {\overline{S}}$ for which

8 $$\begin{aligned} 0<A_{i_{1},j_{1},3}\; if\; i_{1}<j_{1} \; or \; { 0< }A_{i_{1},j_{1},1}\; if\; j_{1}<i_{1} \end{aligned}$$

moreover

9 $$\begin{aligned} 0<A_{i_{2},j_{2},1}\; if\; i_{2}<j_{2} \; or \; { 0< }A_{i_{2},j_{2},3}\; if\; j_{2}<i_{2}. \end{aligned}$$

Then, fixing m$_{1}=0,$ the maximizer of the likelihood function exists and is unique.

Roughly spoken, Theorem 1 requires that a player from group *S* beats a player from group ${\overline{S}}$ and vice versa.

For general distribution, sufficient conditions for the existence and uniqueness of (6) are provided in publication (Orbán-Mihálykó et al. 2019b) for the case of more than two options. In the following, we formulate the statement for three options:

Let us define the graph $G_{TMM}$ as follows: let the vertices be the teams (players, objects) and let the nodes *i* and *j* ($i<j$) be connected, if 0 < $A_{i,j,2}$ or 0 < $A_{i,j,1}\cdot A_{i,j,3}.$ We note that this graph is a part of $G_{c}$ defined above: all edges in $G_{TMM}$ are contained in $G_{c},$ but some of the edges of $G_{c}$ may not be contained in $G_{TMM}$.

#### Theorem 2

(Orbán-Mihálykó 2019b) Let $$F\in {\mathbb {F}}$$. Moreover, suppose that there is a pair (i$$_{1} $$,j$$_{1}$$), i$$_{1}$$ < j$$_{1}$$, for which

10 $$\begin{aligned} 0<A_{i_{1},j_{1},2} \end{aligned}$$

and a pair (i$_{2}$,j$_{2}$), i$_{2}$ < j$_{2}$, for which

11 $$\begin{aligned} 0<A_{i_{2},j_{2},1}\cdot A_{i_{2},j_{2},3}. \end{aligned}$$

If the graph $G_{TMM}$ is connected, then, fixing $m_{1}=0,$ the likelihood function attains its maximum and the maximizer is unique.

Conditions of Theorems 1 and 2 are sufficient but not necessary conditions. To support this statement, we present two simple examples that prove that these conditions do not even cover each other.

#### Example 3

Let there be n=3 elements to rank, $A_{1,2,2}=A_{1,3,2}=A_{2,3,1}=A_{2,3,3}=1,$ all the other elements $A_{i,j,k}$ are zero. Then the condition of Theorem 1 does not hold (see *S*=$\left\{ 1\right\} ,$ ${\overline{S}}$=$\{2,3\}$) but the assumptions of Theorem 2 do.

**Fig. 1:**
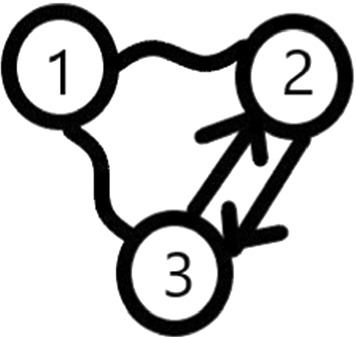
The graph of Example 3 (${{}^{\sim }}$ is for draw; -> is for win)

The graph of Example 3 can be seen in Fig. 1.

#### Example 4

Let there be n=3 elements to rank, $A_{1,2,1}=A_{1,3,2}=A_{1,3,3}=$A$_{2,3,1}=1,$ all the other A$_{i,j,k}$ are zero. In this case, the graph $G_{TMM}$ is not connected (there is no edge from element 2), but all subgroups satisfy the condition that at least one of the elements wins over at least one elements from the complement. Then the conditions of Theorem 2 do not hold, but the conditions of Theorem 1 do.

**Fig. 2:**
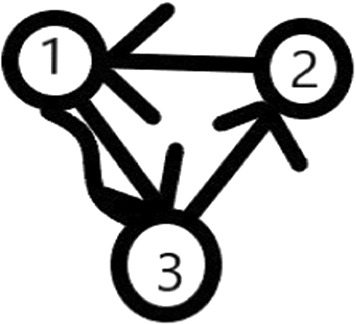
The graph of Example 4 (${{}^{\sim }}$ is for draw; -> is for win)

The graph belonging to Example 4 can be seen in Fig. 2.

Examples 3 and 4 show that the maximizer of (6) can exist and can be unique even in the cases when the conditions of Theorem 1 or those of Theorem 2 are not satisfied.

## Conditions for aggregating separate groups

In what follows, we formulate a generalization of the above theorems. The motivation of such a statement is the intention to create a unified ranking. If there is no link between the separate groups, we may reach a unified ranking on the basis of the scores from the groups with the help of the row sum method. But this ranking might be misleading. If the teams of group $SG_{1}$ are approximately equal in strength, then they all collect moderate scores. If, on the other hand, the teams in group $SG_{2}$ differ significantly in strength, the best may have significantly higher score than the others. If we then interweave the two groups based on these scores, the leader of group $SG_{2}$ will be ranked above that of group $SG_{1}$, even if $SG_{2}$ is the weaker of the two groups. Therefore, the interwoven ranking is not reliable. The same situation may occur in the case of local methods, including the Elo-motivated methods. The situation is different when applying AL and TMM. These methods are global methods, the strengths form a global system. The strength of a team is affected by the results of their opponents’ results with other teams, too. Investigating the problem of interweaving separate groups with the help of TMM, we realized that both theorems (Theorems 1 and 2) require too strict conditions. It is clear that we need at least one comparison between the separate groups, without which it is not possible to reach a unified evaluation. If the result is a tie, then Theorem 2 can be applied but Theorem 1 cannot. If the result is a win, then we also need a defeat, but in many cases the required win and defeat are between different pairs. Therefore, the graph $G_{TMM}$ of the set of the two groups is not connected. Thus, Theorem 2 cannot be applied. From the mathematical point of view the phenomenon can be explained by the fact that the likelihood function depends only on the differences of the expectations. If we evaluate the groups separately, we can fix one parameter in both groups. But if we evaluate the unified system, we can fix only one parameter.

Now we formulate Theorem 5, which contains a sufficient condition for the possibility of preparing a unified ranking in case of $s=3$ options based on a minimal number of matches between the separate groups.

### Theorem 5

Let $F\in {\mathbb {F}}$. Suppose that the objects to rank $(1,2,\ldots ,n)$ are separated into two nonempty disjunct subgroups ($D_{1}$ and $D_{2}$) and for both subgroups the conditions of Theorem 2 are satisfied. Suppose that there exists an element $i_{3}$ in group $D_{1}$ and an element $j_{3}$ in group $D_{2}$ for which

12 $$\begin{aligned} 0<A_{i_{3},j_{3},1}, \end{aligned}$$

moreover, an element $i_{4}$ in group $D_{1}$ and an element $j_{4}$ in group D$_{2}$, for which

13 $$\begin{aligned} 0<A_{i_{4},j_{4},3}, \end{aligned}$$

or there exists an element i$_{5}$ in group D$_{1}$ and an element $j_{5}$ in group $D_{2}$ for which

14 $$\begin{aligned} 0<A_{i_{5},j_{5},2}\text {.} \end{aligned}$$

Then, fixing $m_{1}=0,$ the likelihood function of all comparisons achieves its maximal value and its argument is unique.

**Fig. 3:**
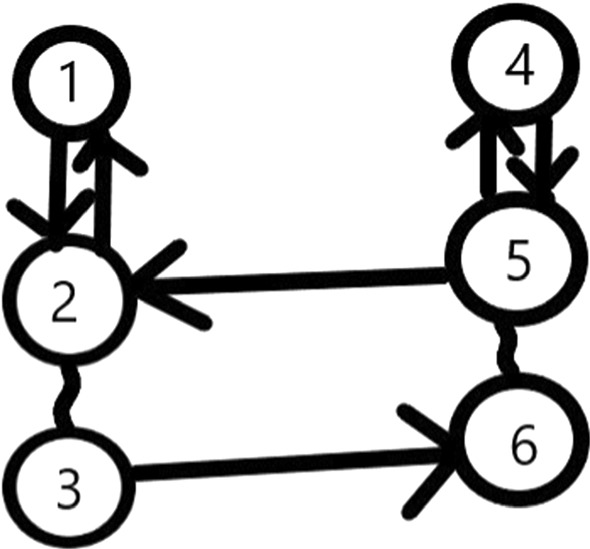
The graph of Example 6 (${{}^{\sim }}$ is for draw; -> is for win)

The proof of Theorem 5 can be found in Appendix A. We note that a similar statement can be made for more than two groups interconnected by (12) and (13) or by (14) as a chain. Note that $$i_{3}$$ is not necessarily different from $$i_{4}$$ and $$j_{3}$$ is not necessarily different from $$j_{4}.$$ They can be either different or equal. In Theorem 2, the equality of the pair ($$i_{3},j_{3})$$ and ($$i_{4} ,j_{4})$$ is required. In the following, we bring an example where neither the conditions of Theorem 1, nor those of Theorem 2 are satisfied, but the conditions of Theorem 5 hold (see Fig. 3).

### Example 6

Let $A_{1,2,1}=$ $A_{1,2,3}=1,$ $A_{2,3,2}=1,$ $A_{2,5,1}=1,$ $A_{3,6,3}=1,$ $A_{4,5,1}=$ $A_{4,5,3}=1,$ $A_{5,6,2}=1,$all the other A$_{i,j,k}$ are zero. The reader can check that $S=\left\{ 1,2,4,5,6\right\}$ and $\overline{S}=\left\{ 3\right\}$ do not satisfy (8) and (9). The graph G$_{TMM}$ is not connected, as there is no edge between the subsets $\left\{ 1,2,3\right\}$ and $\left\{ 4,5,6\right\}$. The conditions of Theorem 2 hold for $SG_{1}=\left\{ 1,2,3\right\}$ and $SG_{2}=\left\{ 4,5,6\right\} ,$ moreover, there is a win from both subgroups towards the other (see Fig. 3).

Finally, we provide a counterexample in which the global methods (TH, BT and AL) work properly when interweaving separate groups, but the point-based evaluation does not.

### Example 7

Let us have two groups, with 4–4 teams. Group X contains elements E,F,G and H. Group Y contains elements I,J,K and L. In the group phase, in Group X every teams plays with each other twice, and the same is true for Group Y. The results of the matches in groups are the following: $A_{E,F,2}=A_{E,F,3} =1,A_{E,G,3}=A_{E,H,3}=2,$ $A_{F,G,2}=A_{F,G,3}=A_{F,H,2}=A_{F,H,3}=1,$ $A_{G,H,1}=A_{G,H,3}=1,$ $A_{I,J,1}=A_{I,J,3}=1,$ $A_{I,K,2}=A_{I,L,2} =A_{J,K,2}=A_{J,L,2}=2,$ $A_{K,L,1}=A_{K,L,2}=1.$ The links between the groups are $A_{E,L,1}=A_{E,L,2}=1.$ All the other elements of data matrix A are zero.

**Fig. 4:**
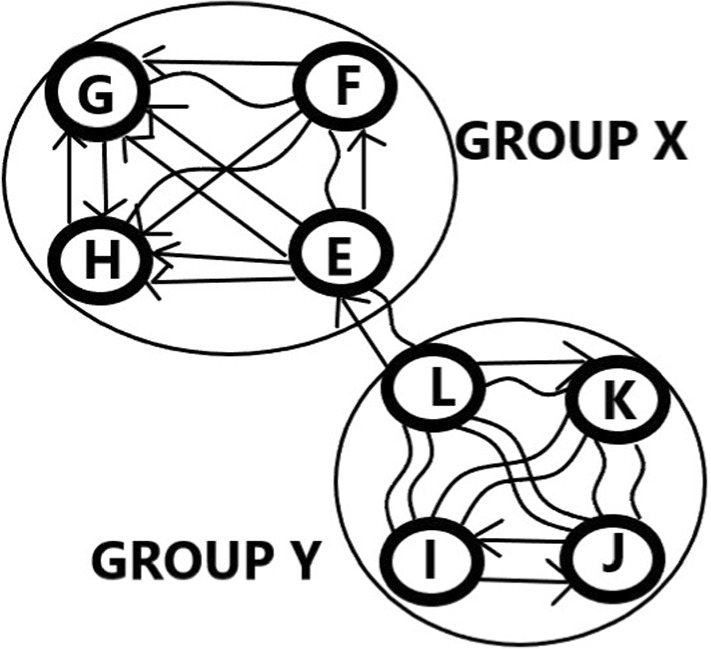
The graph belonging to Example 7. The results of the comparisons of Group X, Group Y, and the connections (${{}^{\sim }}$ is for draw, -> is for win)

The data of Example 7 can be seen in Table 1 and are also demonstrated in Fig. 4.

**Table 1:** Results of Example 7

GROUP	Pairs	Loss	Tie	Win
X	E–F	0	1	1
X	E–G	0	0	2
X	E–H	0	0	2
X	F–G	0	1	1
X	F–H	0	1	1
X	G–H	1	0	1
Y	I–J	1	0	1
Y	I–K	0	2	0
Y	I–L	0	2	0
Y	J–K	0	2	0
Y	J–L	0	2	0
Y	K-L	1	1	0
X–Y	E-L	1	1	0

The evaluations of the groups by TH, BT and AL are contained in Tables 2 and 3.

**Table 2:** The results of the evaluations of Group X (r.p.: number of points divided by the number of matches played)

Gr.X	Ranking	*TH* $({\widehat{m}}_{i})$	*BT* $({\widehat{m}}_{i})$	*AL* $({\widehat{w}}_{i})$	r.p.
E	1	2.160	1.904	0.446	1.75
F	2	0.985	0.860	0.257	1.167
G	3–4	0	0	0.149	0.75
H	3–4	0	0	0.149	0.75

**Table 3:** The results of the evaluations of Group Y (r.p.: number of points divided by the number of matches played)

Group Y	Ranking	*TH* $({\widehat{m}}_{i})$	*BT* $({\widehat{m}}_{i})$	*AL* $({\widehat{w}}_{i})$	r.p.
L	1	0.669	0.660	0.285	1.167
I	2–3	0.335	0.330	0.249	1
J	2–3	0.335	0.330	0.249	1
K	4	0	0	0.217	0.833

The links are a draw and a defeat between the first team of Group X and the first team of Group Y. The information is the following: the best team of Group Y is stronger than the best team of Group X, as once L beats E, once they have tie. This information is reflected in the evaluations by TH, BT and AL.

**Table 4:** The interwoven ranking of Group X and Y. (r.p.: number of points divided by the number of matches played)

Gr X,Y	Ranking	*TH* $({\widehat{m}}_{i})$	*BT* $({\widehat{m}}_{i})$	*AL* $({\widehat{w}}_{i})$	r.p.	Ranking
L	1	3.736	3.424	0.208	1.25	2
I	2–3	3.465	3.172	0.182	1	4-5
J	2–3	3.465	3.172	0.182	1	4–5
K	4	3.193	2.921	0.158	0.833	6
E	5	2.656	2.426	0.120	1.5	1
F	6	1.244	1.139	0.070	1.167	3
G	7–8	0	0	0.040	0.75	7–8
H	7–8	0	0	0.040	0.75	7–8

TH, BT and AL work in the same way: element L becomes the strongest and pulls upward all the elements of Y. The elements of Group X follow them in their original ranking (see Table 4). Due to the different numbers of matches played, we calculated the relative points, i.e. the number of points divided by the number of matches. The points are calculated as follows: win deserves 2 points, tie 1 and loss 0 point. In the ranking based on the relative points, the first team is E, and it is ranked above L, which is a strange ranking. The points from the groups dominate the ratio and the new results could not be integrated into the ranking sufficiently. Conclusion is the same in the case of the generalized row sum method for any value of $\varepsilon$, and in the case of least squares method, too (Csató 2021a).

## Concatenating separate groups in EHF Women’s Champions League

In this section, we present the possibilities of Theorem 5 on the real data of EHF Women’s Champions League 2020/2021. The results of the matches can be found on the web-page https://ehfcl.eurohandball.com/women/2020-21/matches/ (Data 2021).

First, the matches were played in two separate groups. The conditions of Theorem 2 are fulfilled in both Groups A and B. The evaluation results, rankings and ratings for Groups A and B by TM, BT and AL are included in Tables 5 and 6, respectively. The teams are listed in official rankings, column “r.p.” contains the ratios of the points achieved in the group stage and the number of matches played. “r.” denotes ranking. Comparing these results, we note that the results of TH, BT, AL and relative points are different from the official (point-based) ranking in the case of Group A. This is partly due to the allocated points, i.e., that some matches were cancelled because of the Covid-19 pandemic, and, for example, CSM Bucuresti was allocated points twice without playing matches. In Group A, the rankings provided by all three methods and also by r.p. are the same, although the calculated strengths are different.

**Table 5:** The evaluations of Group A’ s results ( r. p.: ratios of the points and number of matches played)

Teams		*TH*		*BT*		*AL*	
In official rankings	r.p.	r.	${\widehat{m}}_{i}$	r.	${\widehat{m}}_{i}$	r.	${\widehat{w}}_{i}$
1 Rostov-Don	1.584	1	0.535	1	0.495	1	0.197
2 Metz Handball	1.384	2	0.360	2	0.281	2	0.165
3 CSM Bucuresti	1.084	5	$-0.052$	5	$-0.119$	5	0.128
4 FTC-Rail Cargo Hungaria	1.142	4	0	4	0	4	0.134
5 Vipers Kristiansand	1.272	3	0.132	3	0.042	3	0.148
6 Team Esbjerg	0.770	6	$-0.397$	6	$-0.386$	6	0.097
7 RK Krim Mercator	0.538	7	$-0.712$	7	$-0.728$	7	0.076
8 SG BBM Bietigheim	0.250	8	$-1.311$	8	$-1.270$	8	0.057

**Table 6:** The evaluations of the Group B’ s results ( r. p.: ratios of the points and number of matches played)

Teams		*TH*		*BT*		*AL*	
In official rankings	r. p.	r.	${\widehat{m}}_{i}$	r.	${\widehat{m}}_{i}$	r.	${\widehat{w}}_{i}$
1 Győri Audi ETO KC	1.714	1	1.210	2	1.098	1	0.222
2 CSKA	1.642	2	1.172	1	1.141	2	0.208
3 Brest Bretagne Handball	1.308	3	0.536	3	0.517	3	0.153
4 Odense Handbold	0.928	4	0	4	0	4	0.105
5 Buducnost	0.858	5	$-0.179$	5	$-0.041$	5	0.098
6 SCM Ramnicu Valcea	0.600	6	$-0.400$	6	$-0.341$	6	0.086
7 BV Borussia Dortmund	0.584	7	$-0.525$	7	$-0.466$	7	0.079
8 HC Podravka Vegeta	0.154	8	$-1.546$	8	$-1.546$	8	0.051

In the case of Group B, the rankings of TH, AL, r.p. and the official ranking are the same, but the results of TH and BT are different in the 1st and 2nd places. Nevertheless, Győri Audi ETO KC beat CSKA and they played a draw in the group stage. Later, in Final Four, playing the match for the bronze medal, Győri Audi ETO KC won a spectacular victory over CSKA. These results makes the intuition that Győri Audi ETO KC is stronger than CSKA in strength, therefore, TH and AL seem to be more realistic than BT.

In the following, we turn to the concatenated ranking of the separate groups. If we apply relative scores, the interwoven ranking can be set up without any connection between the groups. But there is no guarantee for a trustworthy unified ranking. If we use TH, BT or AL, we need some information (match result) between the groups. In this case, we want to use as small piece of information as possible. As there was no tie in the play-offs, we need at least two match results to connect the groups by TH and BT. First, let us take into consideration the results of the matches between the best and worse teams in Groups A and B. The best team of Group A, Rostov-Don beat HC Podravka Vegeta (last team in Group B). Similarly, Győri Audi ETO KC (best team of Group B) beat SG BBM Bietigheim (last team in Group A). In this case, neither the condition of Theorem 1, nor that of Theorem 2 hold. To prove that consider the subgroups $SG_{1}$, the teams of Group A together with Győri Audi ETO KC and its complement, $SG_{2},$ the teams of Group B without Győri Audi ETO KC. As Győri Audi ETO KC had only victories and draws in the group stage, there is no win from $SG_{2}$ to $SG_{1}$. Consequently, the conditions of Theorem 1 are not satisfied. For the same reason $G_{TMM}$ is not connected, therefore, Theorem 2 can not be applied. It is easy to check that the conditions of Theorem 5 are satisfied, therefore, the evaluation can be performed anyway in case of TH and BT. AL also works, as the graph $G_{c}$ is connected. The unified ranking with the estimated strengths can be seen in Table 7. The parameter of the last team is fixed to 0. The rankings by TH and BT differ from each other on the first and second place. As BT ranks CSKA above Győri Audi ETO KC even in the group stage, hence the interwoven ranking by BT is consistent.

**Table 7:** The interwoven rankings, ratings and weights using the wins of the bests against the worsts (in Italic: the teams of Group A)

Teams	*TH*			*BT*			*AL*	
	r.	${\hat{m}}_{i}$	${\hat{w}}_{i}$	r.	${\hat{m}}_{i}$	${\hat{w}}_{i}$	r.	${\widehat{w}}_{i}$
Győri Audi ETO KC	1	2.689	0.154	2	2.536	0.144	1	0.109
CSKA	2	2.654	0.148	1	2.576	0.150	2	0.103
*Rostov-Don*	3	2.301	0.103	3	2.203	0.103	3	0.098
*Metz Handball*	4	2.108	0.086	5	1.963	0.081	4	0.083
Brest Bretagne Handball	5	2.032	0.080	4	1.979	0.081	5	0.076
*Vipers Kristiansand*	6	1.878	0.069	6	1.715	0.063	6	0.075
*FTC-Rail Cargo Hungaria*	7	1.743	0.060	7	1.675	0.061	7	0.067
*CSM Bucuresti*	8	1.688	0.056	8	1.546	0.053	8	0.064
Odense Handbold	9	1.521	0.048	9	1.495	0.051	9	0.052
Buducnost	10	1.343	0.040	10	1.444	0.048	10	0.049
*Team Esbjerg*	11	1.331	0.040	11	1.265	0.040	11	0.048
SCM Ramnicu Valcea	12	1.127	0.032	12	1.162	0.036	12	0.043
BV Borussia 09 Dortmund	13	1.011	0.029	13	1.049	0.032	13	0.039
*RK Krim Mercator*	14	1.010	0.029	14	0.904	0.028	14	0.038
*SG BBM Bietigheim*	15	0.390	0.015	15	0.334	0.016	15	0.029
HC Podravka Vegeta	16	0	0.011	16	0	0.011	16	0.026

The rankings of TH and AL coincide, and there are only two differences (1-2 and 4-5) between the rankings of TH and BT. The similarity of the rates were measured by Garuti compatibility index (Garuti 2020). The estimated expectations were transformed to the interval (0,1) by

15 $$\begin{aligned} \underline{{\widehat{w}}}=({\widehat{w}}_{1},\ldots ,{\widehat{w}}_{n})=(\frac{\exp ({\widehat{m}}_{1})}{\sum _{i=1}^{n}\exp ({\widehat{m}}_{i})},\ldots ,\frac{\exp ( {\widehat{m}}_{n})}{\sum _{i=1}^{n}\exp ({\widehat{m}}_{i})}), \end{aligned}$$

as in Orbán-Mihálykó et al. (2019a, 2019b). The inverse transformation is

16 $$\begin{aligned} \underline{{\widehat{m}}}= & {} ({\widehat{m}}_{1},\ldots ,{\widehat{m}}_{n})=(\log ( {\widehat{w}}_{1})-\underset{i=1,2,\ldots ,n}{\min }(\log ({\widehat{w}} _{i})),\ldots , \log ({\widehat{w}}_{n})\nonumber \\{} & {} -\underset{i=1,2,\ldots ,n}{\min }(\log (\widehat{ w}_{i})). \end{aligned}$$

Table 8 contains the Spearman and Kendall rank correlations (Zar 2005; Kendall 1938), moreover Garuti compatibility indices of the interwoven rankings by TH, BT and AL using the results in the groups and the wins of the bests against the worsts. Both Spearman and Kendall rank correlations demonstrate that there are only small differences in rankings. One can see that the Garuti index between TH and BT is above 0.95, which means that these ratings are compatible. On the other hand, Garuti index between AL and the others are under the level 0.9, which means that the ratings of AL and TH are not compatible. The same can be stated for AL and BT.

**Table 8:** Spearman and Kendall rank correlations of the teams’ rankings, and Garuti compatibility indices of the teams’ ratings after the aggregation, using the wins of bests against the worsts

	Spearman			Kendall			Garuti		
	*TH*	*BT*	*AL*	*TH*	*BT*	*AL*	*TH*	*BT*	*AL*
*TH*	1	0.994	1	1	0.967	1	1	0.951	0.822
*BT*	0.994	1	0.994	0.967	1	0.967	0.951	1	0.840
*AL*	1	0.994	1	1	0.967	1	0.822	0.840	1

It is an interesting question whether it would be enough to use the result of a single match only from the above-mentioned two matches or not. If we omit one match, Theorems 1, 2 and 5 do not work. TH and BT can not be applied. With the help of theoretical arguments one can prove that the maximal value of the likelihood function is not reached. The likelihood function is strictly monotone increasing in a certain direction. Table 9 contains the rankings and the weights provided by AL evaluations. AL–I uses only the win of Győri Audi ETO KC against SG BBM Bietigheim, but not the result of the match between Rostov-Don and HC Podravka Vegeta. By contrast, AL–II uses the win of Rostov-Don against HC Podravka Vegeta, and not the result of the match between Győri Audi ETO KC and SG BBM Bietigheim. AL–0 uses both matches. The evaluations can be performed, as the graph $G_{c}$ is connected in all three cases. The result is unexpected: the strengths decrease for the group from which the winner is taken, and they increase for the group of the loser. The result can be explained as follows: if Győri Audi ETO KC beats SG BBM Bietigheim, the AL method indicates this result by including a multiplier 3 in the AHP matrix. However, as Table 7 shows, this ratio is actually larger than 3; it may be closer to 4. This explains the decrease in weight for the winner team. However, it is an apparent contradiction: if Győri Audi ETO KC has one more win, and the win of Rostov-Don is not taken into consideration, the performance of Győri Audi ETO KC becomes worse and that of Rostov-Don becomes better, and vice versa.

Similar phenomenon has already been demonstrated in the literature (Chebotarev and Shamis 1999; González-Díaz et al. 2014). In our case, we have presented in a live case, that the phenomenon may appear if we concatenate separate groups. This example supports that it is worth being careful with AL while concatenating even if the method works theoretically.

**Table 9:** The interwoven rankings and ratings of the teams of Group A and Group B using both matches as links (AL–0), using only the victory of Győri Audi ETO KC (AL–I) and using only the victory of Rostov-Don (AL–II) (in Italic: the teams of Group A)

Teams	*AL* − 0		$AL-I$		$AL-II$	
	r.	${\widehat{w}}_{i}$	r.	${\widehat{w}}_{i}$	r.	${\widehat{w}}_{i}$
Győri Audi ETO KC	1	0.109	2	0.096	1	0.125
CSKA	2	0.103	4	0.089	2	0.117
*Rostov-Don*	3	0.098	1	0.112	4	0.086
*Metz Handball*	4	0.083	3	0.094	5	0.072
Brest Bretagne Handball	5	0.076	8	0.066	3	0.086
*Vipers Kristiansand*	6	0.075	5	0.084	6	0.065
*FTC-Rail Cargo Hungaria*	7	0.067	6	0.076	8	0.058
*CSM Bucuresti*	8	0.064	7	0.073	9	0.056
Odense Handbold	9	0.052	10	0.045	7	0.059
Buducnost	10	0.049	12	0.042	10	0.055
*Team Esbjerg*	11	0.048	9	0.055	13	0.042
SCM Ramnicu Valcea	12	0.043	13	0.037	11	0.048
BV Borussia 09 Dortmund	13	0.039	14	0.034	12	0.044
*RK Krim Mercator*	14	0.038	11	0.043	14	0.033
*SG BBM Bietigheim*	15	0.029	15	0.032	16	0.025
HC Podravka Vegeta	16	0.026	16	0.022	15	0.029

**Table 10:** Spearman and Kendall rank correlations and Garuti compatibility indices of the teams’ ratings after the aggregation AL–0, AL–I and AL–II

	Spearman			Kendall			Garuti		
	AL–0	AL–I	AL–II	AL–0	AL–I	AL–II	AL–0	AL–I	AL–II
AL–0	1	0.938	0.971	1	0.817	0.883	1	0.876	0.875
AL–I	0.938	1	0.853	0.817	1	0.700	0.876	1	0.766
AL–II	0.971	0.853	1	0.883	0.700	1	0.875	0.766	1

All three index types show that the largest distance is between AL–I and AL–II. This is natural, as the data which were used for these evaluations, contain information contrary to each others. Concerning the evaluation methods, Garuti indices represent larger differences compared to the rank correlations. According to the Garuti indices, all evaluations are incompatible, as Table 10 shows.

## The robustness of the aggregation

In this section, we investigate the robustness of the interwoven results. We analyse the variability of the results, i.e. the ratings and the rankings, as the function of the match results used for connecting the groups. To do that, as a base, we consider weights and the ranking based on all matches played in the group stage and the play-offs. It can be easily checked that, as there is a win and a loss between a pair of teams in different groups during the play-offs, the graphs $G_{TMM}$ and $G_{c}$ are connected, therefore, all three methods provide unique evaluation results. These are contained in Table 11.

**Table 11:** The rankings and ratings of the teams using the matches played in the groups and the play-offs (in Italic: the teams of Group A)

Teams	*TH*			*BT*			*AL*	
	r.	${\widehat{m}}_{i}$	${\widehat{w}}_{i}$	r.	${\widehat{m}}_{i}$	${\widehat{w}}_{i}$	r.	${\widehat{w}}_{i}$
Győri Audi ETO KC	1	2.626	0.160	1	2.468	0.145	1	0.114
CSKA	2	2.368	0.124	2	2.336	0.128	2	0.099
*Rostov-Don*	3	2.191	0.104	3	2.106	0.101	3	0.093
Brest Bretagne Handball	4	2.070	0.092	4	1.999	0.091	4	0.085
*Metz Handball*	5	1.967	0.083	5	1.879	0.080	5	0.080
*Vipers Kristiansand*	6	1.693	0.063	6	1.601	0.061	6	0.069
*FTC-Rail Cargo Hungaria*	7	1.574	0.056	7	1.569	0.059	7	0.063
Odense Handbold	8	1.516	0.053	8	1.496	0.055	9	0.057
*CSM Bucuresti*	9	1.506	0.052	10	1.436	0.052	8	0.059
Buducnost	10	1.365	0.046	9	1.445	0.052	10	0.053
SCM Ramnicu Valcea	11	1.186	0.038	11	1.204	0.041	11	0.047
*Team Esbjerg*	12	1.103	0.035	12	1.112	0.037	12	0.043
*RK Krim Mercator*	13	1.071	0.034	14	0.986	0.033	14	0.041
BV Borussia Dortmund	14	0.999	0.032	13	1.040	0.035	13	0.041
*SG BBM Bietigheim*	15	0.291	0.016	15	0.303	0.017	15	0.028
HC Podravka Vegeta	16	0	0.012	16	0	0.012	16	0.027

The Spearman and Kendall rank correlations of the results of the evaluations by TH, BT and AL based on all matches in groups and play-offs are contained in Table 12. All three rankings are different but there are small differences among them. Garuti indices in Table 12 are rather different. Between TH and BT, the Garuti compatibility index is above 0.9, but between BT and AL and between TH and AL they are bellow 0.9.

**Table 12:** Spearman and Kendall rank correlations of the teams’ rankings, and Garuti compatibility indices of the teams’ ratings after the aggregation by different methods based on the matches in groups and play-offs

	Spearman			Kendall			Garuti		
	*TH*	*BT*	*AL*	*TH*	*BT*	*AL*	*TH*	*BT*	*AL*
*TH*	1	0.994	0.994	1	0.967	0.967	1	0.950	0.836
*BT*	0.994	1	0.994	0.967	1	0.967	0.950	1	0.868
*AL*	0.994	0.994	1	0.967	0.967	1	0.836	0.868	1

The expectations in Table 11 make it possible to estimate the probabilities of the possible results of the matches played in play-offs between the best and the last teams applying the formulas (3), (4) and (5) and the estimated values of the expectations ${\widehat{m}}_{i}$ and parameter ${\widehat{d}}.$ For the calculations, we have computed these probabilities by TH and BT, as well, and their averages are contained in the second column of Table 13. If we denote the win of Team C against CC by W, the draw by D and the loss by L, the possible results of the two matches between Rostov-Don and HC Podravka Vegeta, as well as between Győri Audi ETO KC and SG BBM Bietigheim are (W, W), (W, D), (D, W), (D, D), (D, L), (L, D), (L, W), (W, L), (L, L). Table 13 includes the Spearman correlation coefficients of the different rankings: TH, BT and AL refer to the evaluation methods, indices A, B, C refer to the ranking to which they are compared. Letter *A* is for the case when the basis of comparison is the ranking of the teams after play-offs using TH, letter *B* is applied when the comparative ranking is computed by BT and letter *C* refers to comparative ranking computed by AL.

**Table 13:** The probabilities of the possible results between the leaders of the groups and the last teams of the other groups and the Spearman rank-correlations using these results for interweaving methods

Result	Prob.	$TH_{A}$	$BT_{A}$	$AL_{A}$	$TH_{B}$	$BT_{B}$	$AL_{B}$	$TH_{C}$	$BT_{C}$	$AL_{C}$
(W, W)	0.9527	0.988	0.988	0.988	0.985	0.985	0.985	0.994	0.994	0.994
(W, D)	0.0114	0.482	0.476	0.729	0.435	0.429	0.691	0.479	0.474	0.732
(W, L)	0.0095	–	–	0.518	–	–	0.471	–	–	0.515
(D, W)	0.0141	0.535	0.532	0.868	0.582	0.579	0.900	0.532	0.529	0.862
(D, D)	0.0002	0.974	0.985	0.979	0.965	0.982	0.974	0.976	0.991	0.985
(D, L)	0.0001	0.900	0.918	0.706	0.874	0.900	0.668	0.897	0.921	0.712
(L, W)	0.0117	–	–	0.603	–	–	0.650	–	–	0.600
(L, D)	0.0001	0.974	0.962	0.838	0.976	0.974	0.871	0.974	0.956	0.829
(L, L)	0.0001	0.976	0.985	0.979	0.965	0.982	0.974	0.976	0.991	0.985

First of all, we can conclude that the probability of the results of those two matches which were used by us for the connection in the previous calculations is more than 0.95. Every other pair has a minimal chance. Investigating the possibilities of interweaving, we can realize that AL works in all cases, because $G_{c}$ is connected. But TH and BT can not be applied in the case of (W, L) and (L, W). (L, W) can be easily explained as follows: if Győri Audi ETO KC beats SG BBM Bietigheim and Rostov-Don suffers a defeat from HC Podravka, then even the last team of Group B is better than the best team of Group A. The unified ranking seems to be definite, but the measure of the differences can not be determined. Similar argumentation is true for the case (W, L). The fact that AL works and TMM does not in these cases can be explained as follows: a win returns as a number in the pairwise comparison matrix in the case of AL and it returns as a relation in the case of TMM. Although AL works also in the cases of (W, L) and (L, W), the correlations are low, and the interwoven rankings differ to a great degree from the rankings of the teams after play-offs.

In the other cases of the possible results, due to Theorem 5, we have unique interwoven evaluation results by TH and BT, as well.

Interesting cases are (W, D) and (D, W). The order of magnitude of the probabilities belonging to these cases is 0.01. In these cases the last team of a group is approximately as strong as the best of the other group. The winner establishes the stronger group, therefore, the teams of the stronger group are ranked above the teams of the weaker group. The rankings of the evaluations follow these observations. This explains the small rank correlation values with the ranking after play-offs.

The remaining cases have probabilities much below 0.01. In two of these ((D, D), (L, L)) the rankings contained in Table 11 coincide well for all three investigated methods, as the rank correlations in Table 13 show. The last two cases (i.e. (D, L) and (L, D)) result in high rank correlations applying TH and BT, but the rank correlations are medium when AL is applied. One can see that the case considered above, namely, the most probable and eventually realized one, provides rankings that are very similar to the evaluations after play-offs by all three methods (rank correlations are above 98%). If we collate the correlation coefficients belonging to the methods TH, BT and AL, we can see that $TH_A$ and $BT_B$ provide 5 high (at least 0.9) and 2 low (below 0.6) cases from the possible 7. $AL_C$ provides 3 high (at least 0.9), 5 medium (between 0.6 and 0.9) and 1 low (below 0.6) cases from the possible 9 cases. We can conclude that, on the long run, TH and BT behave very similarly, but AL is somewhat different.

## Summary

Paired comparison methods can be applied to evaluate the results of sports tournaments taking into account ties as a possibility. The paper focuses on interlacing separate groups. An example was given to showcase that the usual point-based evaluations do not provide trustworthy results. A theorem is proved in the paper, which allows for making a unified ranking of separate groups based on isolated pieces of information and few links between some elements of the groups, using the Thurstone and the Bradley–Terry method with ties. The theorem is a generalization of previously known statements. It requires reasonable conditions to all purposes. If it was weakened regarding the links, Thurstone and Bradley–Terry methods would not operate, and AHP with LLSM may provide false results compared to the reality. We analysed the stability of the results in the function of the link-information and we found good correspondences.

## A Appendix

### Proof

Assumption (14) guarantees that the graph containing all the vertices of $D_{1}\cup D_{2}$ with all the edges defined is connected. Therefore, by Theorem 3.2 in Orbán-Mihálykó et al. (2019b) we can conclude that the maximizer exists and is unique.

If assumption (14) does not hold, then the above mentioned graph is not connected as $$i_{1}$$ may differ from $$i_{2}$$ or $$j_{1}$$ may differ from $$j_{2}.$$ To prove the existence and the uniqueness of the maximum likelihood estimation, we need additional justification. The proof of Theorem 1 (Orbán-Mihálykó et al. 2019a) and also of Theorem 3.2 in the paper (Orbán-Mihálykó et al. 2019b) relies on the idea of bounded and closed subsets of the arguments and the strictly concave property of the logarithm of the likelihood function. If the conditions of Theorem 3.2 in Orbán-Mihálykó et al. (2019b) hold for groups $$D_{1}$$ and $$D_{2}$$, then, in these subsets, the differences $$m_{i}-m_{j}$$ are bounded. As these differences do not contain common indices of $$D_{1}$$ and $$D_{2},$$ we need the connection between the differences. One can easily see that the property $$0<A_{i_{1} ,j_{1},1}$$ implies that $$m_{i_{1}}-m_{j_{1}}$$ can be restricted to a closed set with finite upper bound. Similarly, the property $$0<A_{i_{2},j_{2},3}$$ guarantees that $$m_{i_{2}}-m_{j_{2}}$$ can be restricted to a closed set with finite lower bound. These, together with the boundedness of the differences of the variables in the groups $$D_{1}$$ and $$D_{2}$$, guarantee that the maximum has to be in a closed and bounded subset with respect to all variables $$m_{i} $$. Therefore, the maximum of the likelihood function is achieved.

The uniqueness can be proved by the theory of strictly convex (concave) functions. The strictly concave properties of the logarithm of the likelihood function in all variables are guaranteed through the subsets $D_{1}$ and $D_{2}$, therefore the likelihood function belonging to all comparisons also has a unique maximizer. $\square$
